# 

**Published:** 1883-12-20

**Authors:** 


### *EDITORIAL NOTICES.*

RIDGE'S FOOD.—This is certainly a fine preparation. See the advertisement in this issue without fail, and give this preparation a trial.

WE invite attention to the advertisement of the Frazier Road Cart, as a matter of interest to the Doctor. It is preferable to a sulky because large enough to take a driver or friend with you if you choose.

SURGICAL INSTRUMENTS.—See the advertisement of A. M. Leslie & Co., Surgical Instrument makers, of St. Louis. We regard this establishment as among the very best in the country.

IMPERIAL GRANUM.—See the special advertisement of this excellent article in this issue of our Journal. The testimonials to its excellent qualities as a food preparation are of a high order.

NO MORE UNDERBIDDING.—We note that the Hospital College of Medicine and the Kentucky School of Medicine have entered into an agreement to make no reduction of fees hereafter, except to an agreed number of beneficiaries, not to exceed five in the former and eight in the latter. An example worthy of emulation.

**Rare Chance for a Doctor.**—A practice worth $2,500, with large, comfortable House and office, Barn, Stables, etc., with Fifty acres of Land, No competition; people hospitable and good pay. Will sell or rent on easy terms.

Parties desiring information as to the owner and locality of this desirable property may obtain it by addressing,

Dr. R. C. WORD, Editor Southern Med. Record.

### *PAMPHLETS & BOOKS RECEIVED.*

Index to Transactions of the American Medical Association, Vols. 1—xxxiii. Prepared by Wm. B. Atkinson, M D., Secretary. Philadelphia: Wm F. Fell & Co. 1883.

Physician's Poket Day Book, by C. Henri Leonard, M. A., M. D., Prof. of Medical and Surgical Diseases of Women and Clinical Gynecology in the Michigan College of Medicine, etc.. Detroit, Mich. An excellent Pocket Memoranda. Has blanks, 20 to 40 formulæs, etc., etc., very useful. Price, $1 to $1.25.

Medical Record Visiting List, or Physician's Diary, for 1884, with many special and importaut memoranda—space for 60 patients per week. Very useful to the practitioner. Full and complete. William Wood & Co, Publishers, New York City.

### RECEIPTED.

1882—Drs. J T Lee, to Aug.; R L Seal, to April; B F Darnell.

1883—Drs. R A Shimpoch, A B Loving. A A Hill, T M Beaty, J E Pope, A J Davis, J B Rutland, R B Shelley, J J McGahay, C F Rodgers, Jno Elsner, J D Bass, to Oct.; W B Clement, H T Shiell, F M Fitzhugh, W B Maxwell, W T Foute,

1884—Drs. T L Lallerstedt, R C Little, G McMillan, to Oct.; A H Sellers, A A Davidson; to July· A A Stanley, J H Wysong, W E Jones, to May; H Bruce, J E Gilcrest, to July; Thos Bailey, to July; J A Ryan, to July; E W Robinson, to July; N H Hunter, to July; J C Moody.

